# A New Bioactive Glass‐Based Hybrid Bioink With Enhanced Cell Viability and Antibacterial Properties

**DOI:** 10.1002/adhm.202503722

**Published:** 2026-09-22

**Authors:** Adam C. Marsh, Ehsanul Hoque Apu, Theofania Kleisoura, Romila Manchanda, Valerie Johnson, Ginger Ross, Ioannis Tsamesidis, Eleana Kontonasaki, DeMarcus Bunn, Ahmet Erdem, Neal D. Hammer, Kurt D. Hankenson, Christopher H. Contag, Nureddin Ashammakhi, Xanthippi Chatzistavrou

**Affiliations:** ^1^ Department of Chemical Engineering and Materials Science Michigan State University East Lansing Michigan USA; ^2^ Department of Biomedical Sciences Northeast Ohio Medical University Rootstown Ohio USA; ^3^ Institute for Quantitative Health Science & Engineering (IQ) Department of Biomedical Engineering Michigan State University East Lansing Michigan USA; ^4^ Department of Chemical Engineering Aristotle University of Thessaloniki Thessaloniki Greece; ^5^ Department of Prosthodontics, School of Dentistry Aristotle University of Thessaloniki Thessaloniki Greece; ^6^ Department of Biomedical Engineering Kocaeli University Umuttepe Campus Kocaeli Turkey; ^7^ Department of Microbiology, Genetics & Immunology Michigan State University East Lansing Michigan USA; ^8^ Department of Orthopaedic Surgery University of Michigan Medical School Ann Arbor Michigan USA

**Keywords:** 3D bioprinting (3DBP), bioactive glass, biomaterials, bone, cell viability, extrusion, hydrogel

## Abstract

Recent advances in three‐dimensional bioprinting (3DBP) technology enable the production of customized bone substitutes. However, osteoconductive materials in their particulate phase may damage cells during extrusion resulting in poor cell viability after 3DBP. We developed a cell‐friendly, osteopromotive, and antibacterial‐based bioink. To achieve this, we combined gelatin methacryloyl (GelMA) with an Ag‐doped bioactive glass‐ceramic (Ag‐BaG) on the molecular level with (3‐Glycidoxypropyl) trimethoxysilane (GPTMS) to deliver a GelMA‐AgBaG (GAB) hybrid material and compared its characteristics with GelMA and a GelMA‐AgBaG blend nanocomposite. Elemental homogeneity was observed by scanning electron microscopy (SEM‐EDS) in GAB. Rheological studies exhibited strong shear‐thinning and high storage moduli. The swelling and degradation behavior up to 30 days demonstrated the materials' applicability for short‐term applications. The cytocompatibility of GAB, along with its antibacterial properties, was observed, with only 2% of cell death compared to 5% for the pure GelMA, and about 35% for the blend (GelMA‐Ag‐BaGNPs), according to the post‐extrusion cell viability. Extruded constructs and bioprinted scaffolds showed metabolic activity and cell viability over time, with the cell‐laden GAB presenting improved cell viability when compared to GelMA and the nanocomposite blend. Thus, hybrid material GAB possesses advanced multifunctional characteristics suggesting GAB as attractive biomaterial for tissue regeneration.

## Graphical abstract

Graphical illustration of the production and characterization process, demonstrating the multiple functions of the new hybrid gelatin methacryloyl‐silver‐bioactive glass (GAB) material. Created in https://BioRender.com.

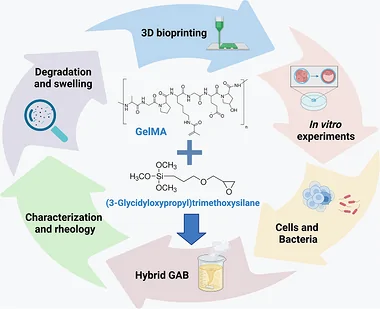

## 1 Introduction

Bone defects represent a major clinical challenge in orthopedic and craniomaxillofacial (CMF) surgery [1, 2]. While autografts remain the gold standard for treating such defects [3], allographs [4] and other biomaterials [5] from natural and/or synthetic sources are viable options. About two million bone graft procedures are performed annually worldwide to treat bone lesions [6]. Autografting is limited by availability, donor‐site morbidity [7], and the difficulty of adapting to the required shape, for example, in the CMF area [8]. The use of devitalized allografts is costly and involves a laborious process [9], resulting in repairs with limited revascularization [10], with risks of immunological reactions [11] and infection [12]. Infected bone often requires secondary revision surgery, which prolongs recovery time and may result in permanent functional loss [13]. Therefore, there is a critical need to develop practical tissue‐engineering approaches to regenerate damaged tissue and combat infection.

An attractive approach is to make biomaterials more biomimetic, seeding cells into scaffolds to facilitate the growth of new tissue constructs [14, 15, 16, 17, 18, 19, 20, 21]. However, many currently available synthetic materials are cytotoxic and therefore not conducive to healing. Naturally occurring biomaterials, such as decellularized tissue matrices [22], collagen [23], and gelatin [24], have been explored for their potential. Gelatin is especially attractive, given that it contains the tripeptide arginyl glycyl aspartic acid (RGD) [25], which is important for cell attachment and function. While these constitute an advance in tissue engineering, an effective cell‐seeded construct remains elusive, as ensuring a homogeneous cell distribution within a scaffold is a challenge that remains unsolved.

The advent of three‐dimensional printing (3DP) technology enables the development of customized bone substitutes, aided by images derived via computed tomography (CT), magnetic resonance imaging (MRI), and computer‐aided design (CAD) [26]. 3DP has been adopted to engineer tissues by including cells into the inks, turning them into bioinks [27].

Unlike conventional 3DP, which typically produces acellular scaffolds without living entities, 3D bioprinting (3DBP) enables the spatially controlled deposition of living cells, biomolecules, and matrix–forming components within the construct. This capability is particularly important in bone tissue engineering, where regeneration depends not only on patient‐specific anatomical requirements but also on the special organization of cells, biological signaling, and microenvironment control. Accordingly, 3DBP is suggested as a very promising technology that offers controlled cell placement, more homogeneous incorporation of biologically active components, and the possibility of fabricating patient–specific living domains with greater biological relevance than conventional 3D printed scaffolds. Therefore, the process of fabricating tissue‐like constructs using cell‐laden bioinks or 3DBP ensures a controlled and homogeneous distribution of cells and any additives within the resulting bioprinted constructs [28, 29]. 3DBP enables the production of patient‐specific constructs that can contribute to improved treatment outcomes. The most commonly used methods for 3DBP are extrusion, inkjet, laser‐based, stereolithography, and microfluidic‐based bioprinting [30]. Among these, the most widely used method is the extrusion‐based technique, which enables the fabrication and use of cell‐infused highly viscous bioinks [27, 31].

Current bioinks used for bone 3DBP usually include osteopromotive elements [32] in the form of calcium‐based ceramics [33, 34] or silicate‐based materials [35, 36] such as bioactive glass (BaG) [36], are commonly blended with other polymeric biomaterials in the form of particles [36] such as bioactive glass (BaG) [36], are commonly blended with other polymeric biomaterials in the form of particles [34, 35, 36, 37, 38, 39]. Conventional composite bioinks cannot ensure a homogeneous distribution of osteoconductive particles. They also carry the risk of particle agglomerations [40], leading to cell damage [32] and death, as it was seen to occur when osteoblasts were loaded into nanocellulose and bioactive glass modified gelatin‐alginate bioinks [41].

Therefore, there is a pressing need to address these problems by developing multifunctional osteopromotive yet cell‐friendly bioinks. We introduce a new bioink biomaterial based on combining various components at the molecular level. This includes gelatin‐based molecules to provide RGD motifs required by cells, methacryloyl components to allow for cross‐linking, bioactive glass to provide osteopromotive function, and silver to convey antibacterial properties. Currently, no report in the literature describes the production of such material that involves the bonding of an inorganic multicomponent osteopromotive material with a polymeric matrix. This new hybrid bioink biomaterial is examined in this paper, demonstrating its printability, antimicrobial properties, and post‐3DBP‐enhanced cell viability.

In addition to its role in bone regeneration, BaG also has anti‐inflammatory and pro‐angiogenic properties [42, 43, 44]. Hence, the BaG component will provide multiple functions: (1) inducing an optimal osteogenic response in cells due to ions that BaG releases following its dissolution [45, 46], (2) enhancing the mechanical properties of the composite scaffolds [47], (3) possessing anti‐inflammatory properties, and (4) promoting angiogenesis. Angiogenesis and osteogenesis are known to occur in tandem during physiological bone healing [48], and therefore, this will be of great importance for the success of any bone regenerative bioink. 3DBP is expected to revolutionize the biomedical engineering field, and innovative bioinks that support cell growth and differentiation lie at the center of this emerging technology.

## 2 Materials and Methods

### 2.1 Material Synthesis

Gelatin methacryloyl (GelMA) was synthesized as reported before [49, 50]. Briefly, type A gelatin (300 bloom; Millipore Sigma) was dissolved in dimethyl sulfoxide (DMSO; Millipore Sigma) at 50°C and stirred at 500 rpm. Methacrylic anhydride (MAA; Millipore Sigma) was added in 1/6 increments every 30 min to achieve a final MAA: lysine ratio of 2.2. The resulting solution was then added to toluene at 3x the reaction volume used to induce precipitation of the GelMA [51]. The solution was then decanted after 24 h, thrice washed with distilled water, and then dissolved in distilled water at 50°C. After dissolution, the solution was placed at 37°C for 24 h to keep GelMA dissolved while allowing for any remaining toluene to evaporate. The solution was then frozen and lyophilized before storage.

To fabricate GAB, the synthesis of GelMA, as described above, and the synthesis of Ag‐BaG following a previously described method [52] were performed simultaneously as separate solutions. Briefly, the Ag‐BaG solution was prepared by adding methanol and mechanically stirring at room temperature, allowing 24 h of stirring, in between reagents, while introducing tetraethyl orthosilicate (TEOS), triethyl phosphate (TEP), aluminum nitrate, silver nitrate, and calcium nitrate (all reagents were obtained from Millipore Sigma). The solvents used were distilled water, 200 proof ethyl alcohol, and methanol. All reagents were used as received without further purification. The photoreduction of silver ions and the evaporation of the organic solvent were prevented by conveniently isolating the beaker in a fume hood and covering it with two layers of parafilm and foil. Then, the coupling agent 3‐glycidyloxypropyltrimethoxysilane (GPTMS) was added to the beaker where the GelMA solution was prepared, after the addition of MAA, to link the organic phase to the inorganic. Next, in the GelMA solution, distilled water and a 3% (*w*/*w*) solution of Ag‐BaG were added to ensure sufficient hydrolysis of the GPTMS and coupling to Ag‐BaG. Mechanical stirring was performed for 24 h at 40°C and 400 rpm to create a homogenized solution. This solution was then precipitated, washed, and lyophilized as previously described in the GelMA synthesis process to prevent changes in material characteristics during storage. Before further use, lyophilized GelMA or GAB was dissolved at 10% (*w*/*v*) in 1X phosphate‐buffered saline (PBS), along with the photoinitiator (VA‐086) at 1.5% (*w*/*v*). The mixture was then photopolymerized at 385 nm for 120 sec, producing hydrogels. The hydrogel samples used for the degradation study were photopolymerized for 70 sec at 405 nm, while optimal printing parameters were achieved for a polymerization time of 25 sec at the same wavelength. In these studies, the hydrogel solution of the samples contained 8% (*w*/*v*) lyophilized GelMA or GAB with the photoinitiator (LAP) at 0.25% (*w*/*v*).

In addition to the comparative study of GAB to GelMA, a comparison to the composite counterpart of GelMA with nanoparticles of Ag‐BaG (GelMA‐Ag‐BaGNPs) was also carried out. These composites consist of a blend of GelMA hydrogel and 3% (*w*/*w*) of nanoparticles Ag‐BaG without the use of a coupling agent. The synthesis of Ag‐BaGNP nanoparticles was previously described by Pajares‐Chamorro et al. [52, 53]. Briefly, solution A was prepared as described above during the synthesis of the inorganic phase in the GAB synthesis. Additionally, in solution B, the catalytic reagents (distilled water, 28–30% ammonium hydroxide, and ethanol) were combined. After calcium nitrate was added to solution A, it was homogenized for 24 h under stirring before pouring solution B into solution A to induce the condensation reactions for nanoparticle nucleation. The concentrations (in molarity, M) of the reagents (methanol, TEOS, H_2_O, NH_4_OH, and ethanol) used are summarized in Table S1. The final suspension was centrifuged at 3000 rpm for 3 min, heat‐treated at 60°C for 6 h, and calcinated at 700°C for 2 h with a 2°C/min heating rate and cooled down to room temperature with 5°C/min (Table S1). Finally, the fabricated Ag‐BaG material (Figure S1) was mortar‐pulverized and washed twice with ethanol to remove calcium‐rich areas and then air‐dried. It should be noted that Ag‐BaG nanoparticles tend to form agglomerates that get monodispersed when they are used in composite synthesis, as described by Pajares‐Chamorro et al. [52, 53]. Particularly, this previous work demonstrates that Ag‐BG nanoparticles agglomerate rather than aggregate, which is crucial for understanding their physicochemical behavior. It was observed that the nanoparticles appear as densely packed clusters; nevertheless, the TEM images show that they remain monodisperse when used in composites with a polymeric matrix. This observation supports the interpretation of agglomeration, in which particles are linked by relatively weak interparticle forces (e.g., van der Waals interactions or electrostatic attraction), as opposed to aggregation, which would imply permanent coalescence and loss of individual particle boundaries.

### 2.2 Lyophilization

The synthesized GelMA and GAB were stored at −80°C and then lyophilized to collect the final materials in a powder form. Specifically, the Labogene Scanvac CoolSafe Basic Freeze Dryer 4 L, with a temperature of −55°C, was used, and the applied pressure and temperature were 0.5 hPa and −50°C, respectively.

### 2.3 Structural, Chemical, and Morphological Characterization

Proton nuclear magnetic resonance (^1^H–NMR) was used to determine the methacrylation of gelatin yielded after performing the GelMA synthesis [54]. The 1D spectra were collected using an Agilent DirectDrive2 500 MHz NMR spectrometer, where a sample concentration of 50 mg∙mL^−1^ in deuterium oxide (D_2_O) was used for all measurements. The degree of substitution (DS) was calculated using the lysine integral method, where the integral of the lysine methylene signals of the gelatin as received was compared to the integral of the lysine methylene signals in GelMA, as described in Equation 1 [55].

(1) $$\mathrm{DSLysine}=(1\;-\;\frac{\int Lysine\;GelMA}{\int Lysine\;Gelatin})\;*\;100$$

To identify the molecular bonds present within the synthesized materials, Fourier‐transformed infrared spectroscopy—attenuated total reflection (FTIR)—was applied using a Jasco FT/IR‐4600 and a PerkinElmer Spectrum 100 IR spectrometer, where spectra were collected from 4000 to 400 cm^−1^. Additionally, spectra relating to the reagents used during the synthesis of GelMA and GAB were collected to utilize FTIR for quality control.

The morphology of the samples was studied using optical microscopy (VHX‐600E Digital Microscope) and scanning electron microscopy (SEM) associated with energy‐dispersive x‐ray (EDX) spectroscopy (SEM‐EDX; Tescan MIRA/JEOL 6610LV and JEOL JSM‐7610F Plus). The morphology and porosity of the lyophilized materials and their hydrogels were studied and analyzed using ImageJ software. Hydrogel samples were prepared for SEM examination by first undergoing a graded series of ethanol dehydration (i.e., x2 in 25%, x2 in 50%, x2 in 75%, x2 in 90%, and x3 in 100% ethanol). The samples were then critical point dried using liquid CO_2_ to preserve the native structure of the materials before being metalized with Os(g) for 15 sec to prevent a buildup of a negative electrical charge. Sample morphologies were captured using a beam voltage of 5 kV. Energy‐dispersive spectroscopy (EDS; Ametek EDAX Apollo X and the OXFORD AZTEC analytical system) was also performed to assess elemental homogeneity using a beam voltage of 20 kV.

The color change of the hydrogel GAB with time was observed with optical images and UV spectra (Ultraviolet and visible light spectroscopy; Lambda900 spectrometer from 340–600 nm with a resolution of 1 nm to determine the chemical status of Ag in the structure) after fabrication and after 60 days of low‐temperature storage.

### 2.4 Rheological Performance Characterization

Rheological characterization was performed (Anton Paar MCR 302 Couette, CTD‐600, SER3 and a BGD 157/TS rheometer with Biuged Instruments Co., Ltd.) on the materials to elucidate their viscoelastic behavior. The storage (G′) and loss modulus (G″) of the GAB hydrogel samples were obtained using temperature sweep (oscillation) as a function of temperature from ambient conditions to 40°C at 1 Hz. The viscosity of the GAB samples was evaluated at 37°C using a shear rate from 0.1 to 100 s^−1^. Moreover, the viscosity of all samples with a temperature in the range of 4°C–37°C was measured at a shear rate of 4 s^−1^.

### 2.5 Printability Studies

Printability was investigated by varying the extrusion pressure (psi), printer bed temperature, extruder temperature (°C), needle gauge, mesh dimensions (mm), printing speed (mm/s), cross‐linking time (s) under UV radiation, and cross‐linking intensity (%) during 3D printing (Allevi 3) (Table S2).

Semi‐quantification of bioink printability was performed according to a previously described procedure [56], where the printability parameter (Pr) is used to quantify similarity with the nature of square‐formed prints. It is based on the circularity (*C*) of the enclosed area, which is defined as *C* = 4𝜋AL^−2^, where *L* is the perimeter and *A* represents the area. The highest circularity is achieved when the *C* = 1 condition is met, representing perfect circularity. When the C value approaches 1, the shape is more circular. The circularity for squared shapes is equal to ¼A rather than that of circles. The Pr is defined as Pr = L^2^/16∙A. The optimal printability is achieved when Pr = 1, indicating that the connected channel forms a complete square shape at the ideal viscosity of the bioink. It is important to note that when Pr > 1, the viscosity during printing was too high, and for Pr < 1, the viscosity was too low during printing. Using ImageJ, optical images of printed constructs were analyzed to determine the perimeter and area of the interconnected channels, enabling the calculation of the perimeter ratio (Pr) of each printed construct (*n* = 15).

### 2.6 Swelling and Degradation Studies

Swelling studies were conducted to determine the swelling ratio by comparing the dry weight (lyophilized) to the weight after immersion in 1X PBS solution. Samples were prepared as 8 mm long and 1.8 mm thick and then incubated at 37°C for 24 h. The weight of swollen hydrogels was defined after removing excess water from the surface. The swelling ratio of hydrogels was calculated according to the formula: (*W*_t_−*W*_0_)/*W*_0_, where *W*_t_ and *W*_0_ represent the weight values of fully swollen and lyophilized (dry) hydrogels, respectively, at each time point with a representative number of nine samples for each time point (*n* = 9) and each hydrogel type.

Degradation studies were carried out by immersing crosslinked hydrogel samples, with a volume of 0.1 mL, a length of 8 mm, and a width of 1.8 mm, in 2 mL of cell culture medium (DMEM; Dulbecco's Modified Eagle Medium, Fisher) at 37°C under shaking at 120 rpm. For the preparation of the crosslinked samples, hydrogels were irradiated at 405 nm for 70 sec with a 2‐watt, 3.2‐volt DC blue light to achieve cross‐linking and stability. Changes in the dimensions of the sample with the immersion time were monitored. The degradation rate of hydrogels is equal to *D*% =$\frac{\;(Wo\;-W\mathrm{tdry})\;}{\;Wo\;}$×100% where *W*_o_ is the initial mass (g) of the hydrogel sample before immersion in medium, *W*_tdry_ is the mass (g) of the sample at the measurement time, after the material has been lyophilized. The sample size of the studied groups was nine at each time point (*n* = 9), with time points at 1, 2, 3, 5, 7, 14, 17, 28, 33, and 35 days. The degradation of the samples was also observed under static conditions with incubation at 37°C. In both cases (static and dynamic conditions), half the medium volume was replaced every 3 days to better simulate the dynamic conditions of the body.

### 2.7 Antibacterial Studies

The antibacterial properties of the GAB hydrogel samples were compared to those of the GelMA hydrogel samples using laboratory‐derived methicillin‐resistant *Staphylococcus aureus* (MRSA) USA300JE2 [57]. MRSA cells were obtained from a frozen stock stored at −78°C, streaked onto tryptic soy agar (TSA), and cultured at 37°C for 24 h to prepare for isolation. 5 mL of sterile tryptic soy broth (TSB) was used to culture a single isolated MRSA colony at 37°C for 24 h under 225 RPM of shaking. 1 mL aliquots of planktonic MRSA were prepared in sterile PBS and normalized to an optical density (OD_600_) of 1, where the MRSA concentration equated to 10^8^ colony‐forming units (CFU) mL^−1^. All cross‐linked hydrogel scaffolds were preconditioned using Dulbecco's modified Eagle media (DMEM) to prevent the osmotic effect [58, 59] from confounding their antibacterial behavior. The MRSA exposed to sample mixtures was then incubated at 37°C for 24 h. Homogeneous aliquots were removed for ten‐fold serial dilutions. Dilutions were plated on TSA and incubated at 37°C to enumerate the CFU and quantify bacterial viability.

### 2.8 Cell Viability and Hemocompatibility Studies

The cytotoxicity of the hydrogels was evaluated by the MTT assay using human periodontal ligament fibroblasts (hPDLFs). hPDLFs were collected from human donors at the Department of Dentistry of Aristotle University of Thessaloniki, as previously described [60], and the study was approved by the Institutional Ethical Committee (110/10‐2‐2021). Cells were cultured in conventional cultivation medium (CCM) consisting of DMEM with 10% fetal bovine serum (FBS) and antibiotics (S/P, 100 U/mL penicillin, 100 mg/mL streptomycin) in an incubator at 37°C, with a humidity level of 95% and 5% CO_2_ for 1, 3, and 6 days. The MTT assay monitors the mitochondrial activity of the cells, and the mitochondrial dehydrogenase can determine their viability. Yellow MTT penetrates only the inner membrane of the mitochondria of living cells, which induces the reduction of yellow mono‐tetrazolium salt to purple formazan crystals. Using a spectrophotometer (Epoch Microplate Spectrophotometer, BioTek), the optical density of each sample was measured at 570 nm with a reference filter of 630 nm, and the values were recorded as a percentage of cell viability compared to the positive control (cells alone). The samples were prepared beforehand, photo‐crosslinked under UV for 70 sec, and then immersed in a nutrient medium for at least 3 days to regulate the pH. Then they were placed in 24‐well plates, and a total of 2 × 10^6^ cells for each sample were added to the wells [61, 62, 63, 64, 65]. Cell viability was studied three times in triplicate for each group at each time point.

Moreover, the Live/Dead assay was applied to study the capability of the samples to be used as cell‐laden bioinks in 3DBP. hPDLFs were cultured to reach the required concentration. Simultaneously, the three types of hydrogels (GelMA, GelMA‐AgBaG nanocomposite, and hybrid GAB) were prepared to ensure a neutral pH. Samples were retained in liquid form, and the extrusion was performed at room temperature. Based on the protocol by Raveendran et al. [66], a cell number equal to 2 × 10^6^ in a total volume of 1 mL was selected for each hydrogel. After collecting the cells, they were stained with two dyes (Ethidium Homodimer – 1 for red and Calcein AM for green), and a volume of 0.2 mL of PBS was added, followed by 0.8 mL of hydrogel. Cell survival immediately after extrusion was studied as well as at four points after extrusion (15, 30, 60, and 90 min). For this purpose, a 0.1 mL sample of each type of cell‐laden hydrogel was extruded and placed for Live/Dead cell counting under a confocal laser scanning microscope (Confocal Microscope Leica DCM 8) to assess the impact of extrusion shear stresses on cell viability [65, 66]. The ImageJ program (National Institutes of Health, Bethesda, MD, USA) was used to quantify the size of live versus dead cells in the collected images.

Hemocompatibility was evaluated in quintuplicates with the tested synthesized biomaterials (GelMA, GelMA Ag‐BaGNP blend, and ‐GAB) after incubation for 24 h at 37°C with a hematocrit of 2%. All the materials were preconditioned as previously described for the MTT assay. Erythrocytes without materials were used as a negative control (Ctrl−), and hemolysis of erythrocytes as a positive control (Ctrl+) was mechanically induced with distilled water. The supernatant of the samples was collected after centrifugation for 5 min at 3000 rpm, and the hemoglobin absorbance was measured at 541 nm, with 700 nm as the reference wavelength.

### 2.9 Adipose Tissue Derived From MSCs

Mesenchymal stem cells (MSCs) were derived from the adipose tissue of adult Sprague Dawley rats. Briefly, abdominal and inguinal adipose tissue was aseptically collected from rats immediately following euthanasia. The tissue was washed twice with PBS and then finely minced under sterile conditions. The minced tissue was incubated with 1% collagenase (Sigma–Aldrich, St. Louis, MO) for 30 min at 5% CO_2_ and 37°C with gentle agitation every 10 min. This suspension was centrifuged at 1050 x G to isolate the stromal vascular fraction, which was subsequently plated in tissue culture flasks in low‐glucose DMEM (Invitrogen/Gibco Carlsbad CA) with 15% FBS (SeraPrime, Fort Collins CO) with 1% antibiotic/antimycotic solution (Invitrogen/Gibco), 1% nonessential amino acids (Invitrogen/Gibco), 1% essential amino acids (Invitrogen/Gibco), and 1% Glutamine (Invitrogen/Gibco). When cells reached 80%–90% confluence, they were detached using 0.25% Trypsin‐EDTA (Invitrogen/Gibco) and either passaged or harvested for use. All cells were used in passages 2–5.

### 2.10 Cell Differentiation Study

To investigate the differentiation of hPDLFs in contact with the synthesized hydrogels, cells were cultured for 28 days in osteogenic differentiation medium (OM), which contained 0.01 µΜ dexamethasone, 50 µg/ml L‐ascorbic acid 2–2‐phosphate, and 10 mM sodium β‐glycerophosphate (Cayman Chemical Company, Michigan, USA). For alizarine red staining (ARS), the cross‐linked hydrogel samples after 3 days of pretreatment were placed in 24 well plates with 2 × 10^4^ seeded cells and 2 mL OM medium per well, while for the ALP activity evaluation in 48‐well plates (ALP) with 10^4^ cells and 1 mL OM per well. On day 0, osteogenic differentiation medium (OM) was added to the wells, and the medium was changed every 2 days. Osteogenic differentiation was assessed after 7, 14, and 28 days. ALP, an early marker of osteoblast differentiation, was evaluated in cell supernatants on day 7 and day 14. The method utilizes p‐nitrophenyl phosphate, which ALP hydrolyzes into a yellow–colored product. On day 28, cells were stained with alizarine red to detect calcium mineralization, a hallmark of osteogenic differentiation. Alkaline phosphate and alizarine red stains were extracted from the samples, and absorbance was measured using a spectrophotometer (Epoch Microplate Spectrophotometer, BioTek) for optical density. The absorbance was read at 405 nm for ALP and 540 nm for alizarine.

### 2.11 Bioink Preparation in the Form of a Single‐Layer 3D‐Printed Mesh

For 3D bioprinting, the concentration of GelMA and GAB was increased to 12% and dissolved in PBS at 70°C in an oven. After dissolution, 0.6% *w*/*v* of the photoiniciator (PI) was added and allowed to dissolve completely in the oven. Once the solution had cooled down to 37°C, the starting cell suspension (10 × 10^6^ cells/ml for GelMA and 50 × 10^6^ cells/ml for GAB) was added to GelMA or GAB solution to achieve a final concentration of 10% *w*/*v* of GelMA or GAB (total volume with cells was 5 mL). To print, we used a secondary syringe with a Luer lock to transfer the liquid GelMA or GAB solution from the glass vial to the printer syringe. The printing parameters for GelMA were: pressure of 1200 mbar, UV (365 nm wavelength), 30% power (100% is 25 mW/cm^2^) for 60 s with a printing speed of 4 mm /second, and it was followed by external Omnicure UV (365 nm wavelength, intensity) exposure for 30 sec (10 mW/cm^2^). All the printing parameters were the same for the GAB samples, except that the pressure was adjusted to 1000 mbar.

### 2.12 In Vitro Biocompatibility of Bioinks in the Form of a Single‐Layer 3D Mesh Using a Live‐Dead Assay

An extruder temperature of 27°C, a 22‐gauge printer needle, and a printing velocity of 4 mm s^−1^ resulted in the successful printing of GAB. Further, 3D bioprinted MSC cell‐laden GAB and GelMA were stained with Live/Dead after 0, 3, and 7 days of culture.

Cell viability was evaluated using Live/Dead Viability/Cytotoxicity Kit (Invitrogen Detection Technologies). The procedure was adjusted in volume for the 6‐well glass‐bottom plates. Calcein AM (4 mM) stock solution was used as a Live (green) stain and diluted to a concentration of 0.5 µL stain per mL PBS. Ethidium homodimer‐1 (2 mM) stock solution was used as a Dead (red) stain and diluted to a concentration of 2 µL stain per mL PBS. The resulting 2 µM Calcein AM plus 4 µM EthD‐1 working solution at 2 mL/well was added directly to cells and constructs for staining. After staining, the stain solution was aspirated from the wells. The wells were then thoroughly rinsed with PBS (1X) three times, and 1 mL of sterile PBS was added to the wells for confocal imaging.

Images were captured on a Leica Stellaris 5 DMI8 confocal microscope and processed with LASX software (both from Leica Microsystems). GelMA constructs were incubated for 45 min at room temperature with stains. GAB constructs were incubated at 37°C for 30 min to account for other living constructs left on the plate during imaging on the confocal microscope. The plate for GAB constructs was maintained at 37°C and 5% CO_2_ in the plating chamber of a confocal microscope during imaging.

Cell viability was quantified using Image J software (Live cells/total cells x 100). Live and dead stained cell quantification was performed in three to four different representative sections of each sample. Mean viability between time points was compared using GraphPad Prism v10, and statistical analysis was performed using a 3‐way ANOVA with multiple comparisons.

### 2.13 Statistical Analysis

Data analysis in cell viability studies was conducted using a two‐way ANOVA followed by Tukey's multiple comparison test in Origin Lab software (OriginLab, 2021, USA). The statistical significance for rheological, degradation, and swelling studies was determined based on the following *p*‐value thresholds: The asterisk (+) used in figures indicates a significant statistical difference for the same group between different time points, and the hashtag symbol (#) denotes a significant statistical difference between different groups at the same time point with a *p*‐value of < 0.05. Also, this symbol (*) marks the statistically significant difference between the different constructs (*p* < 0.05). The data are presented as mean ± standard deviation (SD). All experiments were performed at least in triplicate for each batch, all to ensure reproducibility and statistical reliability.

## 3 Results

### 3.1 Structural, Chemical, and Morphological Characteristics

The ^1^H—NMR spectra of the as‐received gelatin, the synthesized GelMA, and the GAB are demonstrated in Figure 1a. Using Equation 1 and the lysine integral method, the degree of substitution (DS) for GelMA and GAB was found to be ∼100%, providing supporting evidence that the GAB synthesis did not compromise the degree of substitution.

**FIGURE 1:**
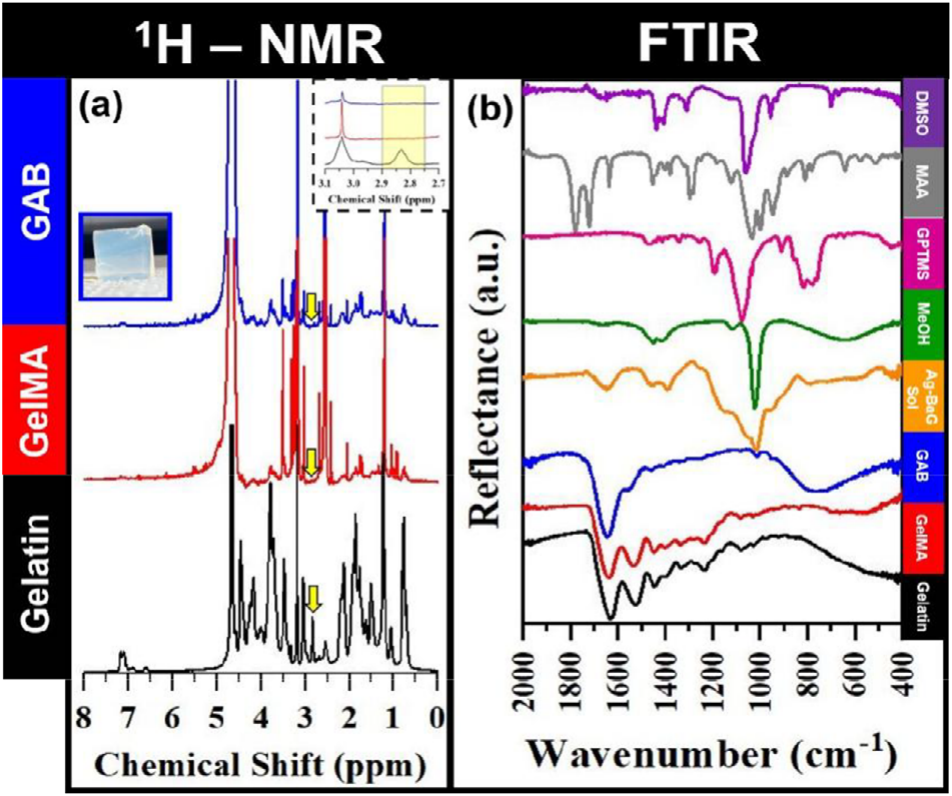
(a) ^1^H—NMR spectra of gelatin (as‐received), GelMA, and GAB. Optical image inserts are also included to show the status of hydrogels after synthesis. The yellow arrows denote the lysine methylene signals used to determine the degree of substitution. A magnified spectrum within the region of interest (ROI) is additionally shown as an insert for clarity. (b) FTIR spectra of the gelatin (as‐received), GelMA, GAB, the Ag‐BaG solution used during the GAB synthesis, methanol (MeOH), the coupling agent (3‐Glycidyloxypropyl) trimethoxysilane (GPTMS), methacrylic anhydride (MAA) used for the methacrylation of the as‐received gelatin, and dimethyl sulfoxide (DMSO) used as the solvent for the synthesis of GelMA and GAB.

Traditionally, GelMA has been synthesized in an aqueous environment such as PBS [67] and using methacrylic anhydride (MAA) as the methacrylation agent, where the maximum achievable DS is observed at ∼80%–85% with significant batch‐to‐batch variations. This was found to be a consequence of using an aqueous environment for GelMA synthesis, as methacrylic anhydride preferentially hydrolyzes in this aqueous environment to form methacrylic acid, which is nonreactive with gelatin. To overcome this and achieve a DS of ∼90%–95%, requires using a 10–32‐fold molar excess of MAA, compared to the lysine groups present in the gelatin [49]. Recently, it has been demonstrated that the use of a carbonate‐bicarbonate system tuned to have a pH of 9.0 has found success in increasing the DS up to 97% while dramatically lowering the MAA required to a 2.2 molar excess [49]; however, a lengthy dialysis post‐processing step was still required.

Here, we aimed to remove the adverse effects created by synthesizing GelMA in an aqueous environment using an organic solvent, DMSO. It was hypothesized that using DMSO would prevent MAA hydrolysis and allow for achieving ∼100% DS during synthesis. Furthermore, it was recently found that the dialysis step could be circumvented when using toluene as a precipitating agent [68]. The small dielectric constant for toluene (*ε* ∼2.4), when exposed to the GelMA solution, increases the attractive forces between the oppositely charged portions of the GelMA, allowing for agglomeration to grow, resulting in the precipitation of the GelMA. An additional benefit of using the toluene precipitation method is that the DMSO and MAA are soluble in toluene, allowing for their removal without the need for a dialysis process.

To verify the effectiveness of the toluene precipitation method, FTIR spectra (Figure 1b) of gelatin as‐received, GelMA, and GAB were collected in addition to the reagents used during the synthesis process of GelMA and GAB. As shown in Figure 1b, minimal spectroscopic changes were observed after the synthesis of GelMA, indicating that the precipitation method used was successful in extracting the GelMA from the solution. Additionally, the characteristic spectroscopic peaks for MAA and DMSO were not observed in the FTIR spectrum of GelMA (Figure 1b), providing further evidence that the precipitation method was successful. For the GAB, the FTIR spectrum (Figure 1b) presented the characteristic amide I, amide II, and amide III groups at ∼1650, ∼1500, and ∼1450 cm^−1^, respectively [69], demonstrating that the GelMA structure was preserved during the GAB synthesis, as expected. The broad Si–O bending peak, located at ∼750–800 cm^−1^ [69] is characteristic of Ag‐BaG, indicating that there was successful incorporation and preservation of Ag‐BaG during precipitation.

Given the successful synthesis of GelMA and GAB, when examining their morphology on the microscopic scale (Figure 2a,b,g,h), apparent morphological differences were observed. The lyophilized GAB (Figure 2g,h) showed thicker filaments of material roughly one micron in thickness with relative uniformity, likely a result of the combination of the organic and inorganic phases at a molecular level. Interestingly, the hybrid system led to a smoother surface morphology of the filaments compared to GelMA alone, where more node‐like features were observed. The pore size of the GAB (Figure 2h) was additionally present on the micron scale. In contrast, the pores for the lyophilized GelMA (Figure 2b) were evident on the sub‐micron scale because of the sub‐micron‐thick filaments.

**FIGURE 2:**
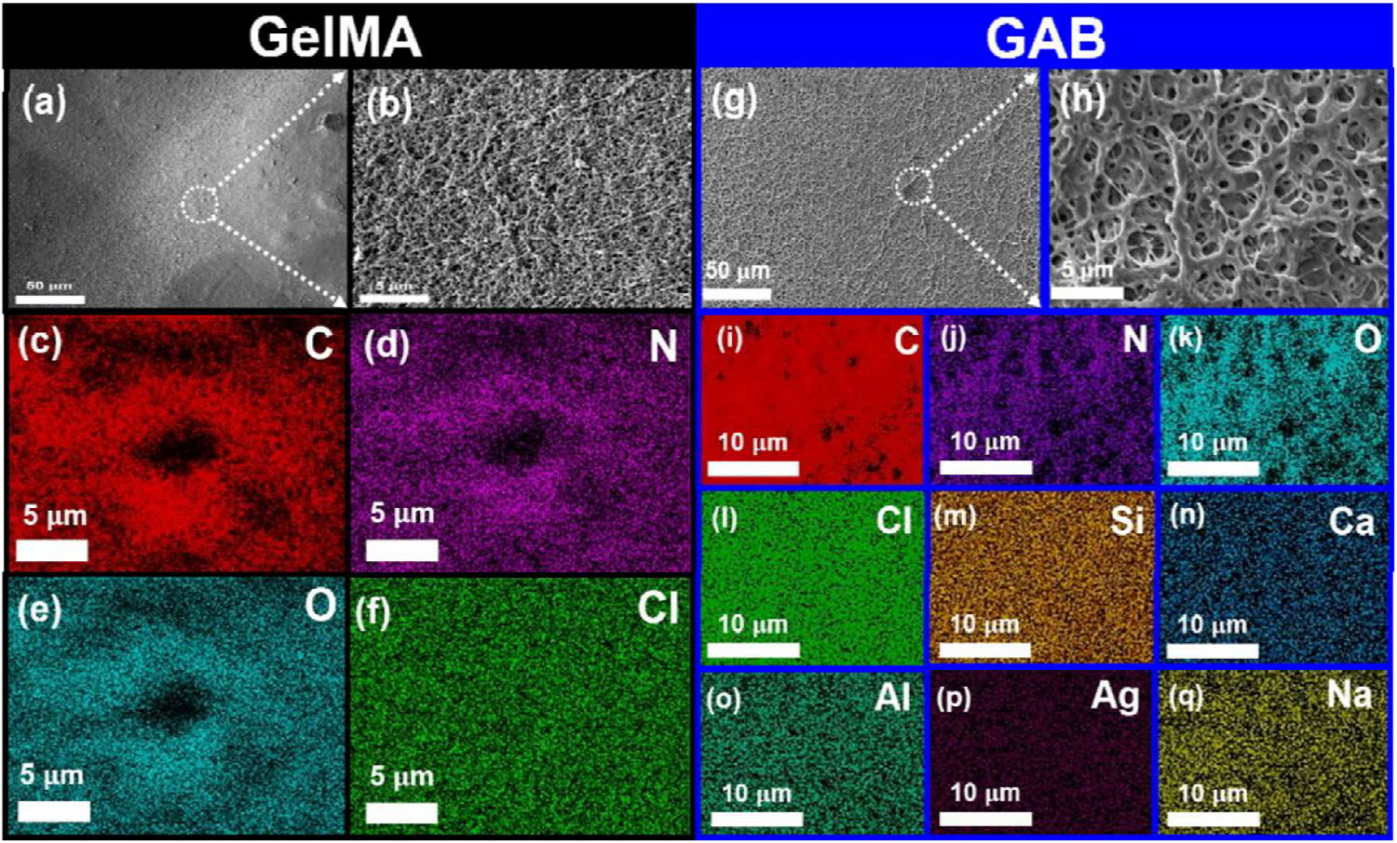
SEM images of (a, b) lyophilized GelMA in addition to the respective EDS x‐ray mapping, where (c) C, (d) N, (e) O, and (f) Cl were all found to be homogeneously distributed down to the micron level. Additionally, SEM images of (g, h) lyophilized GAB and corresponding EDS x‐ray mapping show that (i) C, (j) N, (k) O, (l) Cl, (m) Si, (n) Ca, (o) Al, (p) Ag, and (q) Na are all homogeneously distributed down to the micron level.

Regarding elemental homogeneity, both GelMA (Figure 2c–f) and GAB (Figure 2i–q) were found to have all elements homogeneously distributed down to the micron level. This provides supporting evidence that the synthesis used to deliver GAB was also successful at achieving a high degree of elemental homogeneity. This was critical, as it provides supporting evidence that GAB should exhibit a homogeneous biological response when studied in vivo, allowing for the benefits of both organic and inorganic components to be exhibited simultaneously.

Moreover, the morphological differences between the GelMA and GAB hydrogels, compared to their respective lyophilized samples, are presented in the SEM images in Figure 3. Smoother surfaces are observed, accompanied by a significant reduction in the porosity of the samples to the micron level.

**FIGURE 3:**
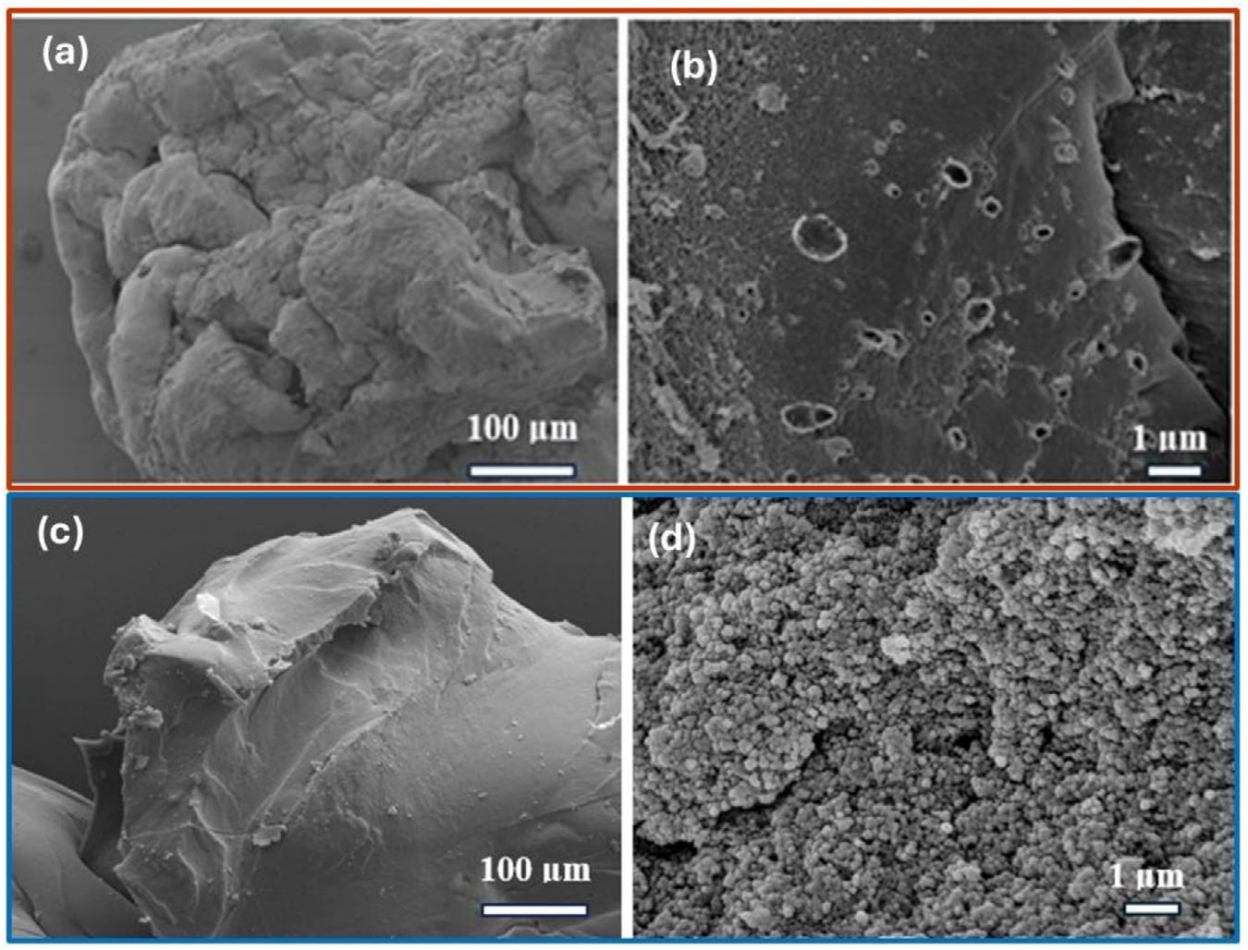
SEM images of (a, b) hydrogel GelMA at low and high magnification, respectively, and (c, d) hydrogel GAB at low and high magnification, respectively.

Additionally, UV spectra and optical images were obtained from the GelMA and GAB hydrogel samples after synthesis and at various storage times, as shown in Figure 4. The UV spectrum of GAB presents a small broad peak in the range of 360–420 nm, which is characteristic of the silver reduction (Figure 4A dark blue line). The intensity of this characteristic peak increases significantly in the UV spectrum of the GAB hydrogel after 60 days of storage at 4°C (Figure 4a light blue line), indicating an increase in Ag reduction with storage time. As such, the UV spectrum of colorless GelMA did not exhibit a peak within this range, as expected (Figure 4a brown line).

**FIGURE 4:**
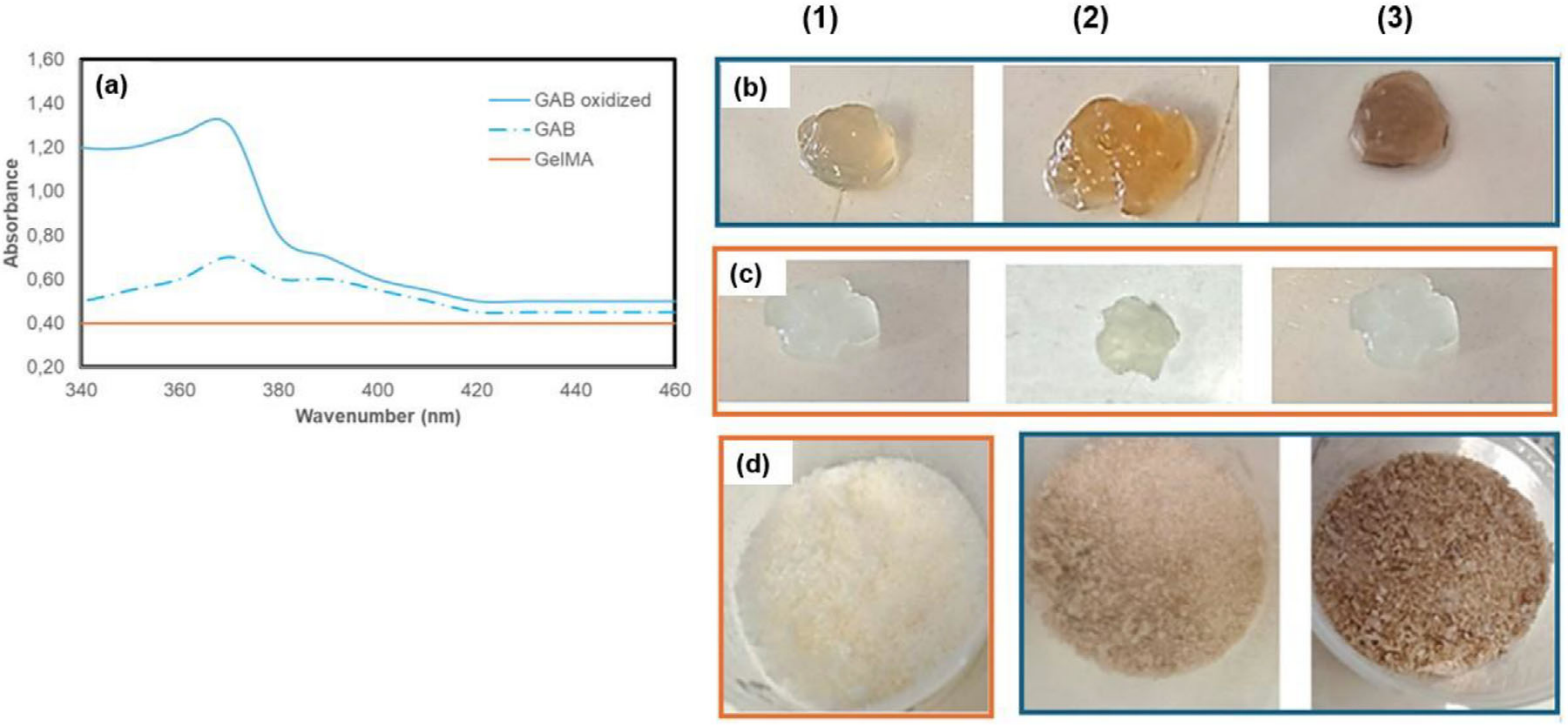
(a) Ultraviolet (UV) spectra of GelMA (brown line) and GAB hydrogels after synthesis (dark blue line) and after 60 days of storage at 4°C (light blue line). (b 1) GAB hydrogel samples at 0 days after extrusion, (b 2) 20 days of storage at 4°C, (b 3) 60 days of storage at 4°C. (c 1) GelMA hydrogel samples in 0 days, (c 2) 20 days of storage at 4°C, and (c 3) 60 days of storage at 4°C. (d 1) lyophilized GelMA colorless and stable with storage time and conditions, (d 2) lyophilized GAB as collected at 0 days of synthesis, (d 3) lyophilized GAB after 9 months of storage at −80°C.

The optical images of the samples also confirmed these color changes. Changes in the color of the synthesized GAB hydrogel samples (Figure 4b1) were observed when the storage time at 4°C was increased to 20 days (Figure 4b2) and 60 days (Figure 4b3). On the contrary, there is no color change in the GelMA hydrogel samples with storage time at 4°C, as confirmed by the optical images in Figure 4c1–3. Likewise, the lyophilized GelMA powder material (Figure 4d1) exhibits no color changes over time. In contrast, the lyophilized GAB powder material, as collected, has a yellowish color (Figure 4d2). However, after 9 months of storage at −80°C, it develops a brownish color (Figure 4d3), similar to the one observed in the GAB hydrogel samples after only 60 days of storage at 4°C. This observation suggests that storage after lyophilization increases its longevity by reducing the rate of silver precipitation; however, further improvements are needed if increased storage longevity is needed.

### 3.2 Swelling, pH, and Degradation Profile

Regarding the performance aspects of GelMA and GAB, in addition to the GelMA‐Ag‐BaG nanocomposite/blend system, the swelling ratio (Figure 5a), remaining mass (Figure 5b), and pH evolution (Figure 5c) were studied. It was found that GAB underwent the least amount of swelling compared to GelMA or the blend GelMA‐Ag‐BaG, although the difference in the swelling ratio between the GelMA‐Ag‐BaG and GAB was insignificant. The presence of the Ag‐BaG inorganic phase in both the nanocomposite and GAB decreased the ability of the material to swell, given that the Ag‐BaG does not experience swelling when exposed to aqueous environments. The swelling ratio (Figure 5a) for GAB was likely decreased compared to the blend GelMA‐Ag‐BaG because of the covalent bonding that existed between the Ag‐BaG and GelMA, as opposed to the weaker Van der Waals interactions between Ag‐BaG nanoparticles and GelMA.

**FIGURE 5:**
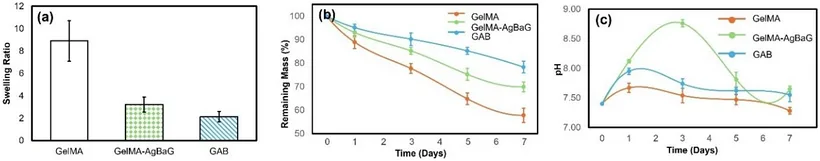
(a) The swelling ratio of GelMA, the blend GelMA‐Ag‐BaG, and GAB. (b) The remaining mass loss for each time point. (c) The pH evolution of GelMA, GelMA‐Ag‐BaG, and GAB after 1, 3, 5, and 7 days of immersion in PBS, in addition to the corresponding.

The GAB was found to be the most stable when the mass loss was studied for up to seven days, compared to the blend, further demonstrating the advantages of covalently bonding the organic phase with the inorganic phase in the hybrid GAB (Figure 5b). Additionally, the pH evolution, studied over seven days (Figure 5c), revealed that GAB exhibited an evolution that closely modeled the pH evolution of GelMA. This was likely due to the covalent bonding present, in addition to the acceptable degree of homogeneity achieved during the synthesis process, which allowed the GelMA to mediate the ion release from the Ag‐BaG more effectively than the blend GelMA‐Ag‐BaG. This displayed a significant increase in pH, which peaked at ∼8.8 before dropping below 8 after 5 days of immersion. Sol–gel‐derived bioactive glasses are known to exhibit a burst release of ions [70] at early stages of immersion, creating an alkaline environment, as shown in Figure 5c. This presents supporting evidence of the benefits that can be achieved when creating the hybrid material GAB.

Moreover, degradation studies were performed under both static and dynamic conditions, for both short and prolonged immersion times, as described above. GelMA hydrogel samples immersed under dynamic conditions exhibited shrinkage and a decrease in length and width, which gradually increased with immersion time and became significant after 20 days of immersion, as presented in Figure 6a1a2.

**FIGURE 6:**
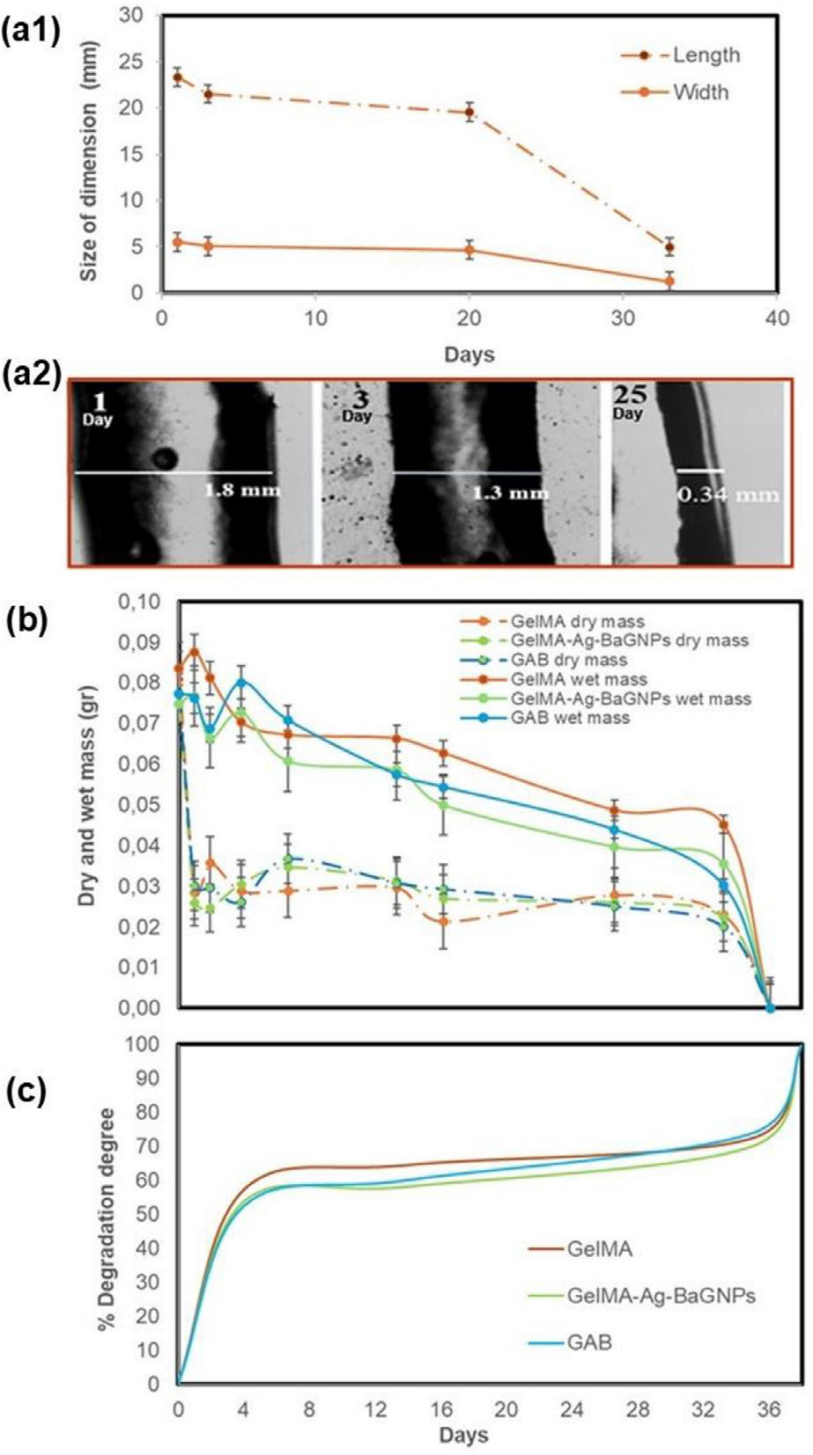
(a1) Degradation study of GelMA hydrogels with dimensional shrinkage under dynamic conditions (a2) Representative optical images of the degradation with time. (b) The evolution of the wet and dry mass of GelMA, blend/composite (GelMA‐Ag‐BaGNPs), and GAB under dynamic conditions. (c) The degradation degree (%) of the hydrogel samples with the immersion time under static conditions.

The decomposition process, under dynamic conditions involving both dry and wet mass, lasted for almost 40 days in all samples (Figure 6b). The dry mass of the samples had a significant mass reduction within the first 5 days of immersion (∼60% reduction), followed by a slower, gradual reduction (less than 10%) for up to 17 days. Then, a more significant reduction (∼25%) occurred in the dry mass of the samples after 35 days of immersion. Similarly, there is a gradual reduction of the wet mass by around ∼55% for all samples when immersed for up to 35 days. Finally, a complete decomposition of all samples was observed after 40 days of immersion. The total decomposition profile between the different sample types is similar. This trend was also evident in the degree of degradation (%) of the crosslinked hydrogels of the samples with increasing immersion time (Figure 6c) under static conditions. All samples showed 60% degradation within the first four days of immersion. An additional degradation of only 10% happened after 30 days of immersion, showing the stability of the samples during this period, with complete degradation observed after 40 days of immersion.

### 3.3 Rheological Properties and Printability

To determine the potential of GAB for applications in 3D printing and 3DBP, its rheological performance was investigated. Table 1 shows a summary of the viscosity of GAB as a function of shear rate, in addition to the storage and loss modulus as compared to values for GelMA obtained from literature, along with the recommended values for each material's property for targeting 3D printing applications. For viscoelastic materials, such as GelMA and GAB, the storage modulus represents the ability of the material to elastically absorb deformation energy, whereas the loss modulus represents the energy dissipated when a force is removed. For GAB, the storage modulus (Figure 7a) was found to be two orders of magnitude greater than either the GelMA reported in the literature or the recommended values (Table 1). The covalent coupling between the organic and inorganic phases in the hybrid GAB enabled the material to absorb a significantly greater amount of deformation energy elastically, providing avenues where the strong ionic bonds from the inorganic phase can contribute to deformation energy storage. The loss modulus for the GAB (Figure 7a) was expectedly increased compared to GelMA, given that the inorganic phase naturally increases stiffness. This is attributed to the orders of magnitude higher stiffness of inorganic bioactive glasses, such as Ag‐BaG, compared to a softer material, such as GelMA.

**TABLE 1:** The experimental rheological properties of GAB and the reported respective values for GelMA hydrogels.

Property	GAB bioink 10% (*w*/*v*)			GelMA bioink 10% (*w*/*v*) literature [71, 72]			Recommended values from literature [27, 38]
Viscosity (Pa^·^s)	40 000	∼1000	∼50	∼300	∼170	∼40	0.3–60 000
Shear Rate (s^−1^)	0.1	1	10	0.1	1	10	
Storage modulus (Pa)	∼75 000			∼800–1000			100–1000
Loss modulus (Pa)	∼20 000			∼5–40			
Shear Thinning	✓✓✓			✓✓✓			✓✓✓

**FIGURE 7:**
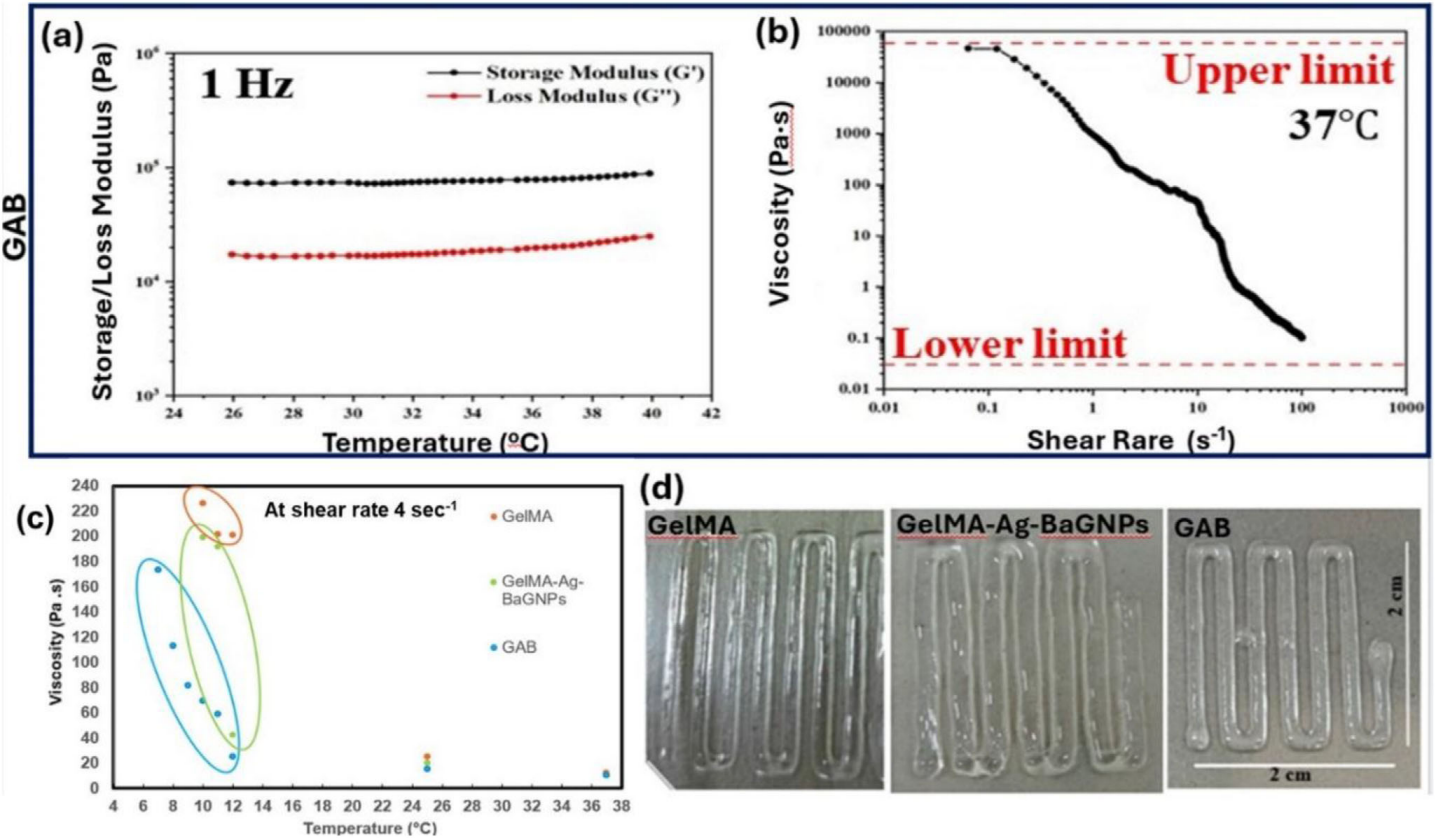
(a) The storage (G′) and loss (G″) moduli for GAB as a function of temperature ranging from 25 to 40°C at 1 Hz of oscillation. (b) The apparent viscosity of the GAB as a function of the shear rate shows that GAB exhibits shear‐thinning behavior. (c) The viscosity of all hydrogel types as a function of temperature at the shear rate of 4 s^−1^. (d) A single mesh layer of 3D printed GelMA, blend GelMA‐Ag‐BaGNPs, and GAB.

The viscosity of the GAB (Figure 7b) was found to decrease as a function of increasing shear rate, an indication of shear‐thinning behavior. The linear region is present between ∼1–10 s^−1^ (Figure 7b). Power fitting was used within the linear region of viscosity (Pa∙s) as a function of shear rate (∼1–10 s^−1^) to determine the flow consistency index (*K*) and flow behavior index (*n*) [31, 73]. $\eta_{\mathrm{app}}=K{\dot{\gamma }}^{\left(n-1\right)}$

GAB was found to have a flow consistency index (*K*) of 417 ± 10 (Pa·s^n^) and a flow index behavior (*n*) of 0.045 ± 0.007 with a correlation coefficient (*R* ^2^) of 0.98 when computed using the linear region, where viscosity decreases as a function of shear rate (i.e., 1–10 s^−1^). The low value of the flow behavior index (*n*) indicates that the GAB hydrogel is a highly shear‐thinning, non‐Newtonian fluid.

Figure 7c shows the viscosity of all hydrogels as a function of temperature in the range of 4°C–37°C with a constant shear rate at 4 s^−1^. When evaluated under ambient temperatures for a specific shear rate, all hydrogels showed a viscosity lower than 50 Pa·s, as supported by both the literature and the observations we noted herein (Table 1). Moreover, all samples showed an increase in viscosity with a temperature drop below 12°C. Finally, all hydrogels exhibited acceptable and successful printability (Figure 7d) when a single‐layer mesh was 3D printed at 12°C for the GelMA and blend GelMA‐Ag‐BaGNPs hydrogels, whereas GAB was printed at 10°C, indicating that the hybrid hydrogel achieves optimal stability and printability at lower temperatures. The rest of the printing parameters were applied as described earlier in Table S2.

### 3.4 Antibacterial Activity of GAB

Dose‐dependent bacterial inhibition was observed for GAB against MRSA. After 24 h of exposure, GAB at 10 mg mL^−1^ showed a statistically significant bacterial inhibition, around 10% reduction compared to the untreated control. The increase in GAB concentration significantly decreases bacterial viability compared to the control, with 15% and 75% reduction for concentrations of 100 and 500 mg mL^−1^, respectively, without, however, observing bactericidal activity, even at concentrations up to 500 mg mL^−1^. There was no bacterial inhibition for GelMA compared to the untreated control, even at higher concentrations (Figure 8). It is expected that longer exposure times would elicit bactericidal behavior.

**FIGURE 8:**
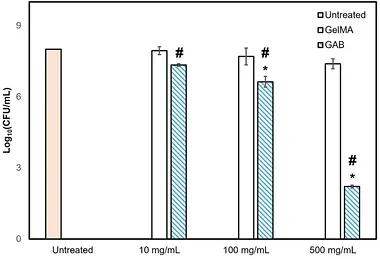
Inhibition of MRSA after 24 h of exposure to different concentrations of GelMA and GAB. (*) *p* < 0.05 comparing samples within the same concentration, (#) *p* < 0.05 comparing the same sample at different concentrations.

### 3.5 Cytocompatibility—Cell Viability—Hemocompatibility of Hydrogel Samples

Cell viability on cross‐linked hydrogel samples was observed for all groups after 1, 3, and 6 days of culture (Figure 9a) with no signs of cytotoxic behavior up to 6 days. Moreover, GAB exhibited 30% higher cell viability after 1 and 3 days of culture, with values significantly exceeding those of all other samples and corresponding controls at each time point. After 6 days of culture, cell viability in GAB samples was around 80% higher, significantly higher compared to pure GelMA and the blend GelMA‐Ag‐BaGNPs samples, although significantly lower (around 50%) compared to the positive control.

**FIGURE 9:**
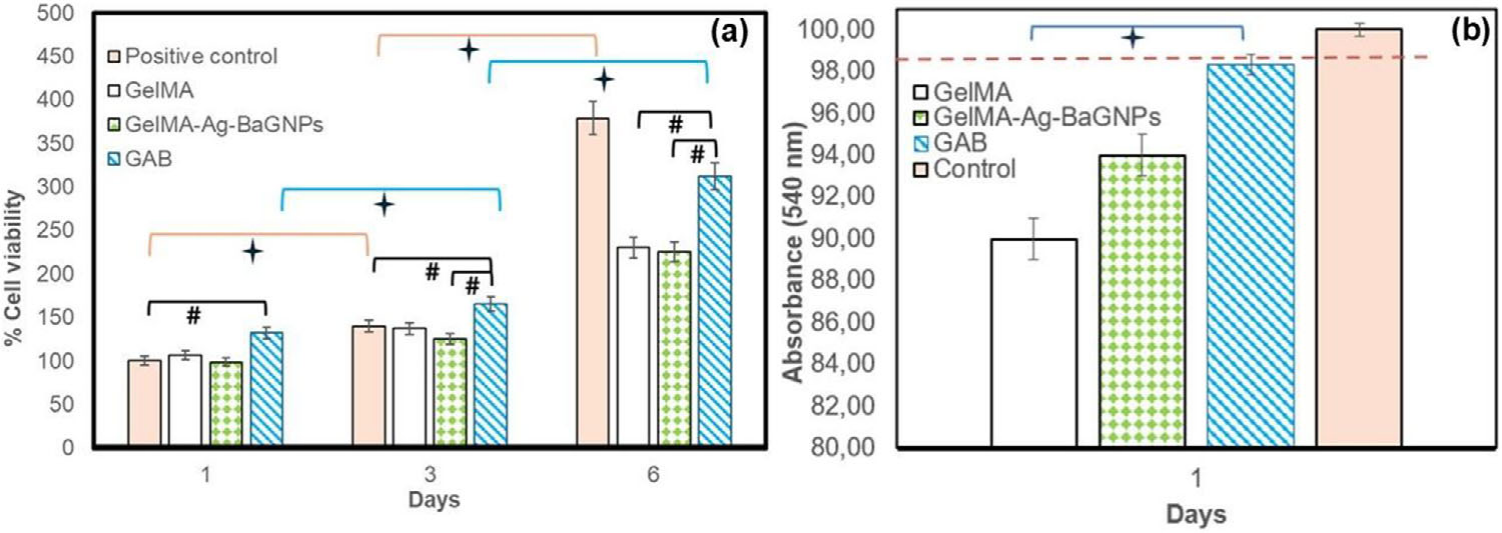
(a) Enhanced cell viability for cells cultured with GAB compared to GelMA and the blend at all time points. (b) Hemocompatibility of cross‐linked hydrogel samples. The asterisk (+) indicates a significant statistical difference for the same group between different time points, and the sharp symbol (#) denotes a significant statistical difference between different groups at the same time point with a *p*‐value of <0.05.

The hemocompatibility of the GAB and blend GelMA‐Ag‐BaGNPs samples was assessed using red blood cell survival, with values of almost 95% for the blend and 98% for the GAB samples. These values meet and exceed the threshold to be characterized as hemocompatible [74]. Interestingly, the hemocompatibility of GelMA samples was reduced, falling below the 95% threshold (Figure 9b).

### 3.6 Cell Differentiation

A significant upregulation of ALP was observed after 7 days of culture in OM with GelMA, blend GelMA‐Ag‐BaGNPs, and GAB samples compared to the control, while GAB and blend showed an additional increase compared to GelMA. While downregulation was observed after 14 days for all samples, GAB showed a significantly lower rate of downregulation compared to the other sample groups evaluated (Figure 10a). The induced cell differentiation was also observed by alizarine red staining, and the secreted calcium minerals at the latest stage of cell differentiation. The measured values were significantly higher for all the hydrogel samples compared to the control. In the case of the blend GelMA‐Ag‐BaGNPs samples, a significantly higher secretion was observed compared to GelMA (Figure 10b).

**FIGURE 10:**
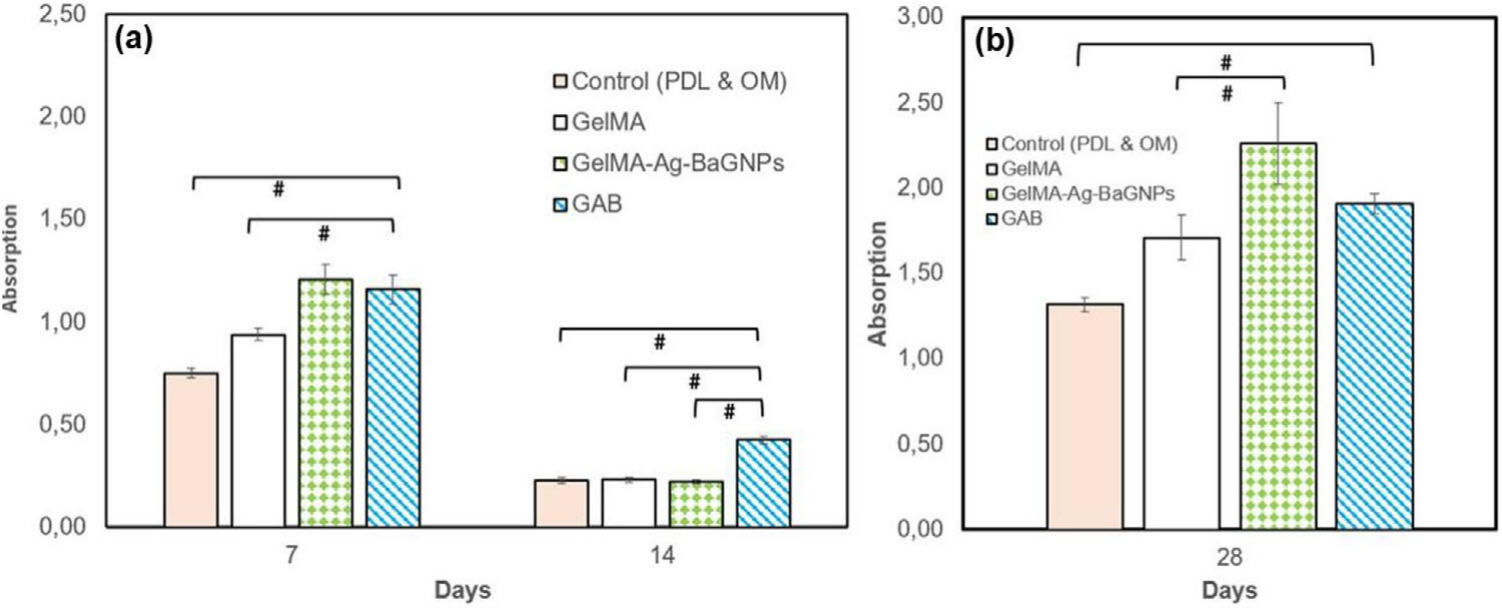
Cell differentiation of PDLs cultured in OM with cross‐linked hydrogel samples (a) ALP expression in the early and late stage of differentiation, (b) alizarine red staining of calcium minerals secreted by differentiated cells. The sharp symbol (#) denotes a significant statistical difference between different groups at the same time point with a *p*‐value of <0.05.

### 3.7 Cell Viability in Cell‐Laden Bioinks After Extrusion

Confocal images revealed a significant difference in cell viability between the GAB and GelMA bioinks compared to the blend GelMA‐Ag‐BaGNPs (Figure 11a). In particular, Dead cells were observed in Live/Dead images to be, on average, at 2% for the GAB and almost 5% in the GelMA, straight after extrusion and up to 30 min (Figure 11b). However, the blend GelMA‐Ag‐BaGNPs bioinks presented a significantly higher number of Dead cells, on average, at 35% after extrusion and up to 30 min.

**FIGURE 11:**
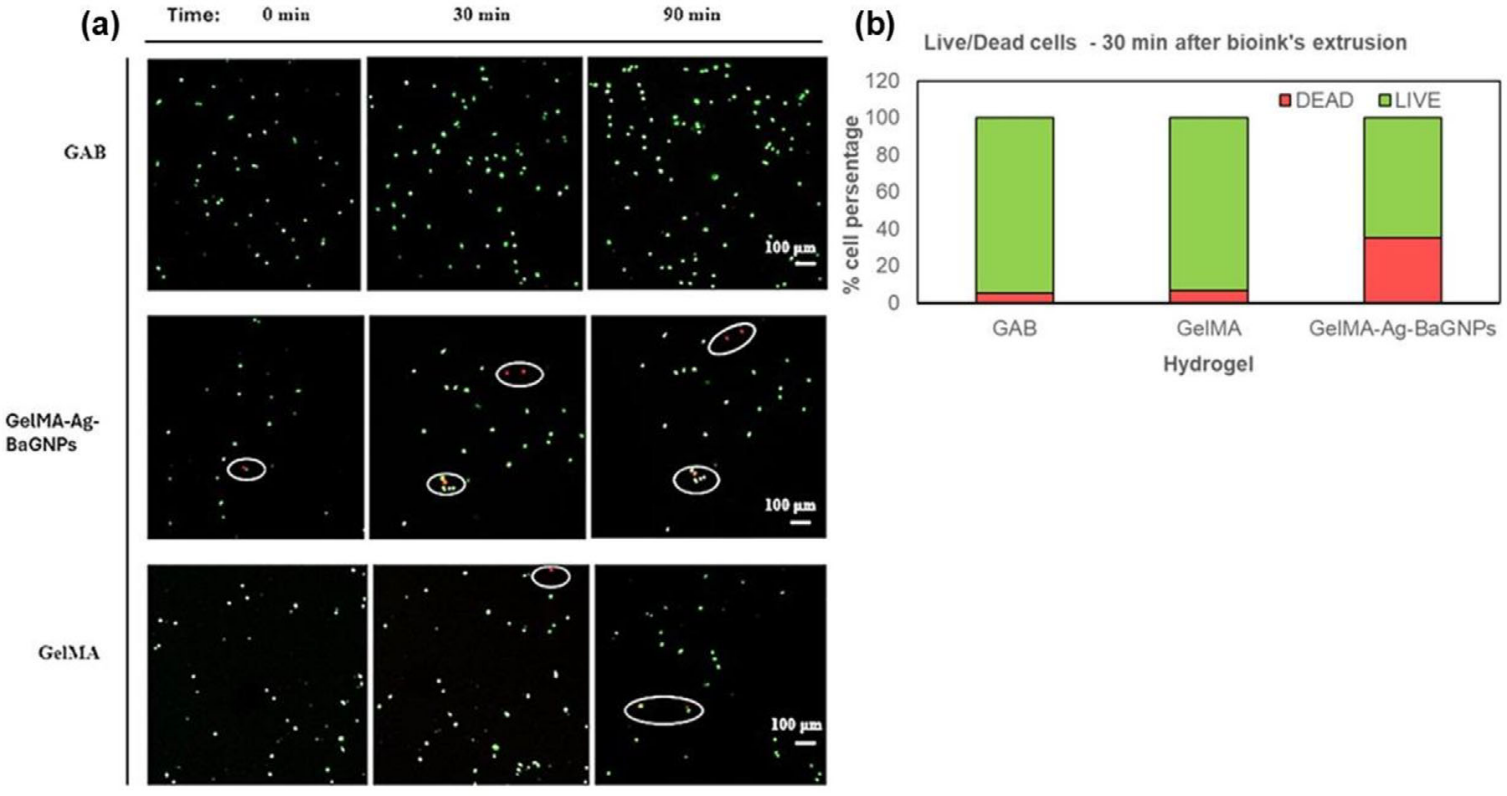
(a) Confocal images of Live/Dead staining of cell‐laden bioinks after extrusion, 30, and 90 min later (Dead cells are marked by the white circles). (b) Calculated percentage (%) of Live/Dead cells in bioinks after 30 min of extrusion.

### 3.8 Cell Viability in Cell‐Laden Bioinks After 3D‐Bioprinting

Metabolic activity of manually extruded MSC‐ and fibroblast‐laden GAB was significantly higher than that of MSC‐ and fibroblast‐laden blend GelMA‐Ag‐BaGNPs (*p* < 0.05). It is also higher than that of MSC‐ and fibroblast‐laden GelMA (Figure 12).

**FIGURE 12:**
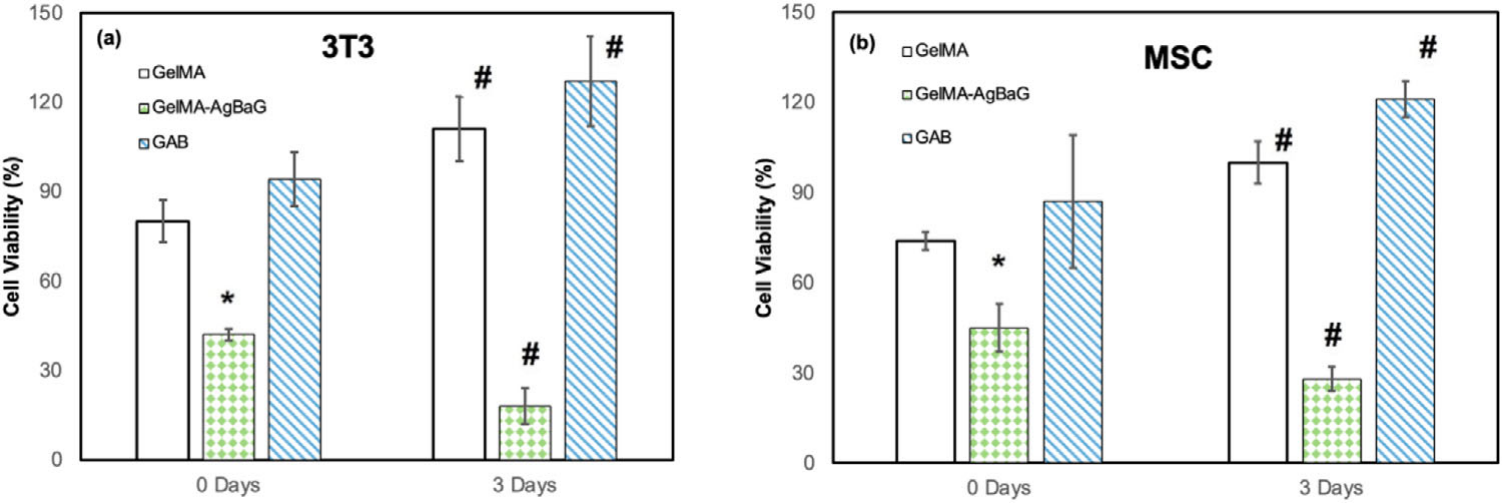
Metabolic activity of MSCs and fibroblasts in extruded 3D GelMA, GAB, and blend constructs. (*) *p* < 0.05 comparing samples within the same time point and (#) *p* < 0.05 comparing the same sample at the two different time points.

A single‐layer mesh was bioprinted using MSC‐laden GelMA and GAB (10 × 10^6^ cells/mL for GelMA and 50 × 10^6^ cells/mL for GAB) as presented in the images of the optical microscope in Figure 13A1 A2 and then cultured in growth medium at 37°C for 7 days. Cell viability, as it was calculated by ImageJ analysis of the confocal images, showed that GELMA constructs had a mean viability of 72% immediately after printing (Day 0), 64% on day 3, and 86% on day 7. Similarly, there was no significant difference in cell viability between day 0 and day 3 in the GAB constructs, with mean values at 64% and 77% for day 0 and day 3, respectively, while day 7 did not show any difference compared to day 3 (Figure 13B1 B2). Confocal images (Figure 13C) revealed that cells remained alive at both time points (3, 7 days) post‐printing in culture, indicating that the 3D bioprinting process did not adversely affect cell viability, while no significant difference was observed across experimental groups during the seven days of culture.

**FIGURE 13:**
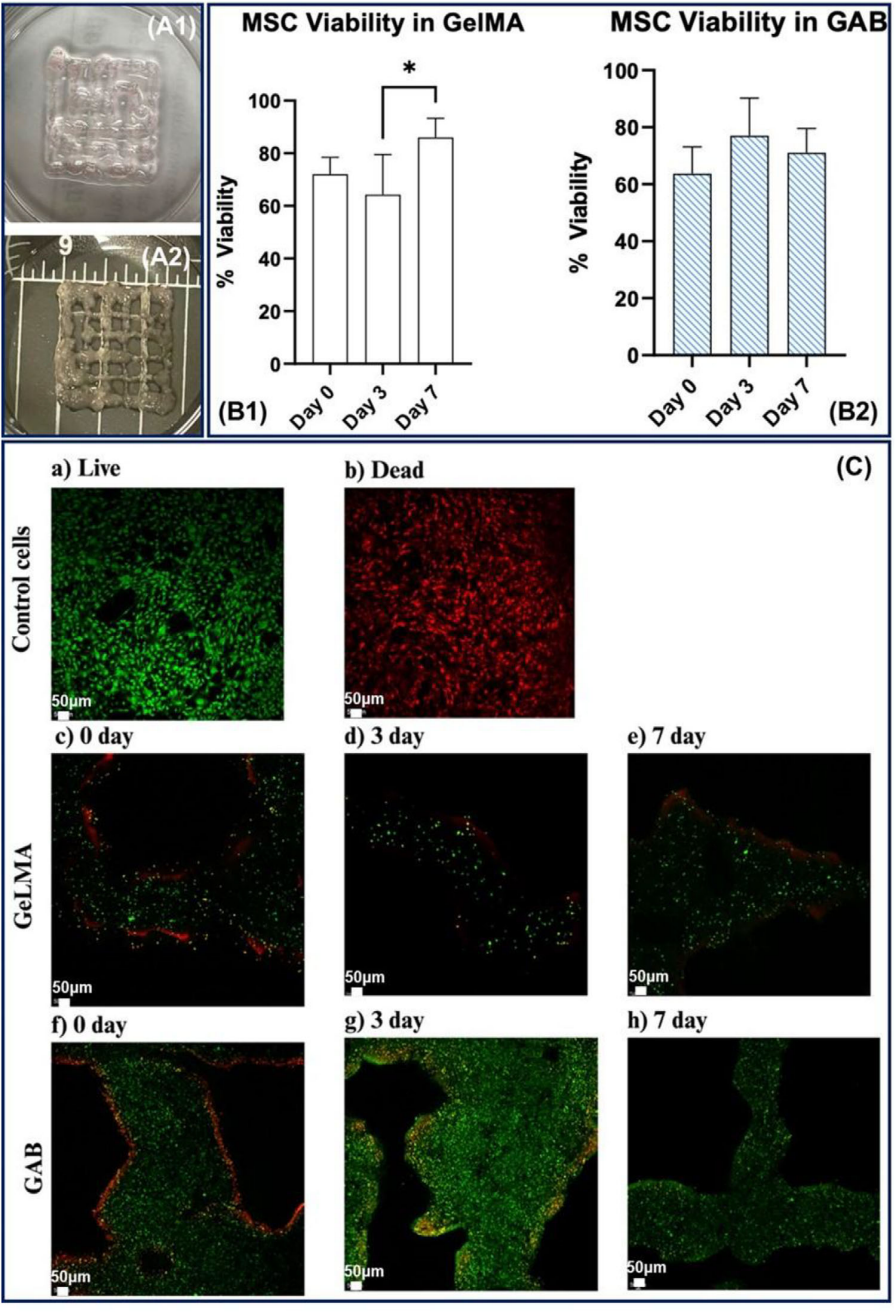
(A1‐A2) Optical images of 3D Bioprinting of MSC‐embedded GelMA (A1) and GAB (A2) grids. (B1‐B2) Quantification of cell viability in GelMA and GAB grids for 3 and 7 days of culture. (*) marks the statistically significant difference between the different constructs *p* < 0.05. Groups were compared using a multiple comparison ANOVA in graph pad prism v10. (C) Confocal microscope images of Live (green)‐Dead (red) stained MSCs in 3DBP GelMA (c, d, e) or GAB (f, g, h) constructs after printing and in culture for 3 and 7 days. Panels (a) and (b) show control groups with MSCs cultured without samples, serving as a baseline comparison.

## 4 Discussion

This study presents the results of the synthesis and performance of modified GelMA–based hydrogels, which have demonstrated enhanced cell support for osteogenic development, 3D printability, rheological stability, and bioactivity, as expected from within the soft biomaterials research field [75, 76].

The structural characterization of GelMA and GAB spectra showed the absence of the characteristic peaks of methacrylic anhydride (MAA) and dimethyl sulfoxide (DMSO) in their FTIR spectra, suggesting successful methacrylateion, precipitation of the solid materials, removal of the organic solvent, and removal of possible impurities in the washing stage during synthesis. Furthermore, the FTIR spectrum of GAB did not show the characteristic peaks for methanol (MeOH) or (3‐glucidyloxypropyl) trimethoxy silane (GPTMS), which confirmed the effectiveness of using toluene for GAB precipitation, and for the removal of the cytotoxic reagents used.

SEM analysis of the GelMA hydrogel revealed that the porous structure remains, albeit with smaller fibers and pores compared to the lyophilized material, due to swelling and the presence of a liquid phase within the hydrogel. The elemental analysis on GAB confirms the existence of elements involved in the synthesis: C, O, N, Si, Al, Na, Cl, and Ca which are homogeneously distributed, while it is noted that the confirmation of the silver (Ag) existence is present only in GAB and justifies its reduction in contrast to GelMA. SEM analysis confirms both the porous structure of the two types of hydrogels and the elemental homogeneity of the samples through EDS x‐Ray mapping. In the case of the GAB hydrogel, the surface is rougher compared to GelMA, and the pores are equally smaller due to the liquid phase.

It is preferential for the GAB to exhibit shear‐thinning behavior as shear thinning allows materials to exhibit self‐healing abilities after removal of the shear forces, allowing for higher quality constructs to be 3D printed [77].

As shown in Figure 7c, the behavior of GelMA followed the known temperature‐viscosity relation for hydrogels. Specifically, an increase in temperature results in a decrease in viscosity and conversely. This behavior explains the change in the physical state of the hydrogel, which is thermosensitive and becomes thinner with increasing temperature, and more gelatinous with decreasing temperature. It is also noted that higher viscosities are associated with better stability for 3D printing and crosslinking of the hydrogel. Evaluating the viscosity values, it appears that the GelMA hydrogel of the optimized formulation (orange dots) exhibits the best printability and is more stable at temperatures above 12°C. The blend/composite GelMA‐Ag‐BaGNPs hydrogel (yellow dots) exhibits equally good printability, with slightly lower viscosity values at 11°C, and shows a significant decrease in viscosity at the higher temperature of 25°C compared to GelMA. However, the addition of nanoparticles has an enhancing effect on the rheological behavior. The hybrid hydrogel GAB (blue dots) exhibits a thinner viscosity, as confirmed by obtaining reduced viscosity values at each temperature compared to the other two types of hydrogels, without compromising printability, which, however, requires lower temperatures. At 37°C, all hydrogels are in liquid form, which makes cross‐linking and printing at this temperature more challenging. It is hypothesized that increasing the concentration of the lyophilized material would increase the viscosity of the resulting hydrogel.

Through elucidation of the optimal combination of parameters, 3D printing of all three types of hydrogels was realized. GelMA with a higher viscosity appears to exhibit the best printing capability at higher temperatures, specifically 12°C and 13°C, and creates stable 3D structures. Regarding the GelMA‐Ag‐BaGNPs blend ink, its printability is also confirmed, with no flow obstruction despite the clogging potential of the homogenously distributed nanoparticles. Additionally, due to its higher viscosity, printability is achieved at slightly higher temperatures, and higher extrusion pressure is required for the same reason. Although the presence of nanoparticles constitutes only a small percentage of 3% wt., it significantly improves the viscosity and printability of the hydrogel and outperforms the hybrid. When printed at slightly lower temperatures, such as 8°C, GAB can achieve a similar print quality as the GelMA‐Ag‐BaGNPs blend.

The viscosity of the GAB Class II hybrid hydrogel is within an acceptable range, given that the applied shear rate is <100 s^−1^. There was a decrease in viscosity as a function of shear rate, indicating shear‐thinning behavior for the GAB Class II hybrid hydrogel. This behavior indicates that the hydrogel will exhibit self‐healing properties after the removal of shear forces.

The ability to 3D print GAB at 15 psi with an extruder temperature only 10 degrees cooler than physiological temperatures is expected to minimize the shear forces and minimize the temperature differential cells would exhibit when incorporated into the material for 3D bioprinting. This study presents supporting evidence of the printability of GAB and demonstrates its potential advantages for 3D bioprinting compared to other hydrogels such as GelMA.

As can be seen from the in vitro degradation, hydrogels are undoubtedly degradable materials. The mass of the hydrogel initially increases as it absorbs part of the liquid medium. However, as the decomposition progresses, both mass and dimensions decrease, with complete mass loss predicted to occur at approximately 33 days under static conditions, following a rapid decrease after 14–20 days. However, even under stirring conditions, with no nutrient refreshing, samples remained for almost 35 days, due to nutrient saturation that slows down the degradation rate. In contrast, renewing the medium every two days resulted in faster degradation at approximately 7–10 days under stirring conditions. In the ideal case of incubation under stirring at 120 rpm, the hydrogel samples are preserved for up to 33 days with the medium refreshed every 4 days. This provides a retention period sufficient to ensure the regeneration of new tissue or the healing of a wound. In general, it is a common aim to maintain hydrogels for at least 14–20 days to enable their effective use for biomedical applications while also demonstrating the ability to degrade into non cytotoxic compounds within 35 days to allow for safe absorption by the recipient. At the beginning of the study, hydrogel swells as it absorbs the nutrient medium; hence, its mass or volume increases as its structure retains the liquid. After this initial stage, decomposition, mass and geometric reductions become the dominant mechanism of interaction. Under the same conditions where GelMA decomposition was examined, the composite and hybrid hydrogels were also examined, showing similar behavior and degradation profiles within a month. As expected, the evolution of both wet and dry mass after lyophilization of hydrogels shows a downward trend until the complete elimination of samples incubated in DMEM. However, static conditions and possible local saturation of the liquid medium lead to an increase in decomposition time. Moreover, a common observation for all three types of hydrogels is swelling up to about day 5, with GelMA showing the highest swelling, while composite and hybrid GAB show less swelling.

Cell viability observed almost 30% higher for cells cultures with GAB compared to the control and the rest hydrogels for cultures up to three days. This enhanced cell proliferation for GAB shows a further significant increase on day 6 compared to the other two hydrogel groups. In particular, cell proliferation in the GAB hydrogel shows an increase of 28% on day 6 compared to the GelMA‐Ag‐BaGNPs blend and 26% compared to the pure GelMA hydrogel. The mechanisms of the antibacterial activity of the Ag‐BaGNPs have been extensively investigated in our previous work. The size of each antibacterial action was quantified, as well as the advantage of these nanoparticles not being toxic to eukaryotic cells, due to their controlled ion‐releasing process, which maintains the concentration of Ag+ ions during the degradation process at a nontoxic range. The hybrid GAB hydrogel may also support a more controlled and localized release environment than freely dispersed nanoparticles, reducing acute exposure of encapsulated cells while still allowing sufficient antimicrobial activity at the material interface.

Additionally, the Live/Dead assay at time zero (straight after extrusion), the hybrid hydrogel GAB shows the highest viability (97.5%). This can be attributed to the reduction of the extrusion stresses. In contrast, the GelMA‐Ag‐BaGNPs blend hydrogel exhibits a viability rate of 82.9% immediately after extrusion, which was 15% lower than GAB. This reduction was likely due to the increased shear stress caused by the presence of nanoparticles. Also, the values measured after 30 min of extrusion were thought to be representative, where GAB was found to be superior having a cell viability of 94.7%. This value was close to the respective measured for GelMA, (93.5% cell viability), while the GelMA‐Ag‐BaGNPs blend showed the highest decrease in cell viability (65%).

Alkaline Phosphatase (ALP) activity and alizarine red staining verified effective cell differentiation. High enzymatic activity shows that the cells successfully underwent an osteoblast‐like transition, generating the enzymes required for matrix maturation. In addition, ARS revealed large, indicating robust extracellular matrix development and mineralization. Active osteogenic differentiation is further supported by elevated alkaline phosphatase (ALP) activity, consistent with the observed increase in calcium deposition. Collectively, these findings demonstrate successful early osteoblast commitment, followed by the maturation of mineralizing bone‐like cells. ARS results show that both the inorganic phase in hybrid GAB and the GelMA‐Ag‐BaGNPs blend enhance osteogenesis, with the latter showing the most pronounced statistical improvement compared to pure GelMA. Finally, confocal results demonstrate that it is possible to build 3D bioprinted cell‐laden GAB constructs, where cells are homogeneously distributed and can survive the printing process as well as remain viable throughout the subsequent culture up to 7 days. These findings highlight the biocompatibility of GAB and support its applicability in 3DBP applications.

## 5 Conclusion

We demonstrated herein the feasibility for synthesizing a hybrid material of GelMA and Ag‐BaG to deliver an advanced multifunctional multicomponent hybrid hydrogel, GAB, where the organic and inorganic phases are molecularly bonded to deliver a novel shear‐thinning biomaterial with unique and interesting capabilities. GAB was demonstrated to be crosslinkable given the inclusion of GelMA, showing that the inclusion of the Ag‐BaG did not compromise crosslinking. This new material undergoes less swelling than blends made of GelMA and Ag‐BaG nanoparticles or GelMA, presenting potentially better stability over time. Degradation of GAB is completed in almost a month, which should be appropriate for engineering of different tissues, allowing gradual replacement by native tissue. GAB has also antibacterial properties inhibiting resistant bacterial strains, MRSA. GAB exhibits a less viscous behavior as compared to its blend counterpart, suggesting the need to investigate this at lower temperatures. A common phenomenon for all the three tested types of hydrogels is their thermo‐reversible nature, the inverse viscosity‐temperature relationship, and the confirmation of their printability with an optimal combination of printing parameters. Finally, the biocompatibility of GAB is confirmed. Furthermore, cell viability of extruded cell‐laden bioinks demonstrates its superiority over to blend counterpart. GAB material enhances osteogenic differentiation, and it is suitable for the 3D bioprinting of MSC‐laden constructs that can be used for bone tissue engineering. Collectively, the results of the present study showed that GAB can be considered an advanced bioink that can be used to advance extrusion‐based 3DBP.

## Funding

This work was funded, in part, by the James and Kathleen Cornelius Endowment at MSU (CHC).

## Conflicts of Interest

The authors declare no conflicts of interest.

## Supporting information




**Supporting File**: adhm71720‐sup‐0001‐SuppMat.docx.

## Data Availability

The data that support the findings of this study are available on request from the corresponding author. The data are not publicly available due to privacy or ethical restrictions.
