# Supplementary material for: A New Bioactive Glass‐Based Hybrid Bioink With Enhanced Cell Viability and Antibacterial Properties
Source: Adv Healthc Mater. 2026 Sep 22;15(39):e03722. doi: 10.1002/adhm.202503722 (PMC13691399; doi:10.1002/adhm.202503722)
Supplement: Supplementary file 1 [file ADHM-15-0-s001.docx]

**A new bioactive glass-based hybrid bioink with enhanced cell viability and antibacterial properties**

Adam C. Marsh^1,^­^#^, Ehsanul Hoque Apu^2,3,#^, Theofania Kleisoura^4^, Romila Manchanda^3^, Valerie Johnson^3^, Ginger Ross^3^, Ioannis Tsamesidis^5^, Eleana Kontonasaki^5^, DeMarcus Bunn^3^, Ahmet Erdem^6^, Neal D. Hammer^~~6~~7^, Kurt D. Hankenson^~~7~~8^, Christopher H. Contag,^3,7^, Nureddin Ashammakhi^3,^*, Xanthippi Chatzistavrou^1,4,^*

**Supplementary**

**Table S1**: Ratios of reagents based on TEOS concentration for the total volume of the reaction after mixing Solution A and Solution B [1].

| Ratios of reagent | **TEOS** | **Methanol/TEOS** | **H_2_O/TEOS** | **NH_4_OH/TEOS** | **Ethanol/TEOS** |
| --- | --- | --- | --- | --- | --- |
|  | 0.23M | 0.02 | 55.9 | 5.3 | 50 |

**Figure S1**: The collected nanoparticles Ag-BaGNPs after synthesis.

**Table S2**. Optimal printing conditions.

|  | Needle gauge | Intensity | Printer bed temperature (^o^C) | Extruder temperature (^o^C) | Mesh (mm) | Mesh density | Extrusion pressure | Extrusion speed (mm/s) | UV & Time | Volume (ml) |
| --- | --- | --- | --- | --- | --- | --- | --- | --- | --- | --- |
| GelMA | 22G | 110% | 14 | 12 | 20x20 | 15% | 155 kPa | 15 | 405 nm & 25 s | 3 |
| Blend  GelMA-AgBaGNPs |  |  | 14 | 12 | 20x20 | 10% | 180 kPa | 20 |  |  |
| GAB |  |  | 10 | 10 | 20x20 | 12% | 35 kPa | 11 |  |  |

GelMA with a higher viscosity appears to exhibit the best printing capability at higher temperatures, specifically 12 ^o^C and 14 °C, and creates stable 3D structures. Regarding the GelMA-Ag-BaGNPs composite ink, its printability is also confirmed, with no flow obstruction due to clogging in the presence of nanoparticles, which were homogeneously distributed. Additionally, due to its higher viscosity, printability is achieved at slightly higher temperatures, and higher extrusion pressure is required for the same reason. Although the presence of nanoparticles constitutes only a small percentage of 3 % wt., it significantly improves the viscosity and printability of the hydrogel and outperforms the hybrid. GAB hybrid hydrogel can be equally printed but is more stable at slightly lower temperatures, such as 10°C.

**References**

[1] N. Pajares-Chamorro, S. Hernández-Escobar, Y. Wagley, P. Acevedo, M. Cramer, S. Badylak, N.D. Hammer, J. Hardy, K. Hankenson, X. Chatzistavrou, Silver-releasing bioactive glass nanoparticles for infected tissue regeneration, Biomaterials advances 154 (2023) 213656.
